# Development of a Machine Learning Model for Predicting Treatment-Related Amenorrhea in Young Women with Breast Cancer

**DOI:** 10.3390/bioengineering12111171

**Published:** 2025-10-28

**Authors:** Long Song, Zobaida Edib, Uwe Aickelin, Hadi Akbarzadeh Khorshidi, Anne-Sophie Hamy, Yasmin Jayasinghe, Martha Hickey, Richard A. Anderson, Matteo Lambertini, Margherita Condorelli, Isabelle Demeestere, Michail Ignatiadis, Barbara Pistilli, H. Irene Su, Shanton Chang, Patrick Cheong-Iao Pang, Fabien Reyal, Scott M. Nelson, Paniti Sukumvanich, Alessandro Minisini, Fabio Puglisi, Kathryn J. Ruddy, Fergus J. Couch, Janet E. Olson, Kate Stern, Franca Agresta, Lesley Stafford, Laura Chin-Lenn, Wanda Cui, Antoinette Anazodo, Alexandra Gorelik, Tuong L. Nguyen, Ann Partridge, Christobel Saunders, Elizabeth Sullivan, Mary Macheras-Magias, Michelle Peate

**Affiliations:** 1 School of Computing and Information Systems, The University of Melbourne, Parkville, VIC 3052, Australia; 2 Department of Obstetrics, Gynaecology and Newborn Health, Faculty of Medicine, Dentistry & Health Sciences, The University of Melbourne, Parkville, VIC 3052, Australia; 3 The Royal Women’s Hospital, Parkville, VIC 3052, Australia; 4 Melbourne School of Population and Global Health, Faculty of Medicine, Dentistry & Health Sciences, The University of Melbourne, Parkville, VIC 3052, Australia; 5 Department of Medical Oncology, Institut Curie, 26 rue d’Ulm, 75005 Paris, France; 6 Gynaecology, The Royal Children’s Hospital, Parkville, VIC 3010, Australia; 7 Murdoch Children’s Research Institute, Parkville, VIC 3010, Australia; 8 Centre for Reproductive Health, Institute for Regeneration and Repair, University of Edinburgh, Edinburgh EH16 4UU, UK; richard.anderson@ed.ac.uk; 9 Department of Medical Oncology, U.O. Clinica di Oncologia Medica, IRCCS Ospedale Policlinico San Martino, 16132 Genova, Italy; matteo.lambertini@unige.it; 10 Department of Internal Medicine and Medical Specialties (DIMI), School of Medicine, University of Genova, 16132 Genova, Italy; 11 Research Laboratory on Human Reproduction, Université Libre de Bruxelles (ULB), 1070 Brussels, Belgium; 12 Fertility Clinic, CUB-Hôpital Erasme, 1070 Brussels, Belgium; 13 Institute Jules Bordet, 1070 Brussels, Belgium; 14 UNICANCER Federation, 75013 Paris, France; 15 Department of Obstetrics, Gynecology and Reproductive Sciences and Moores Cancer Center, University of California San Diego, La Jolla, CA 92093, USA; 16 Faculty of Applied Sciences, Macao Polytechnic University, Macao SAR, China; 17 Department of Breast and Gynecological Surgery, Institut Curie, 26 rue d’Ulm, 75005 Paris, France; 18 School of Medicine, Dentistry and Nursing, University of Glasgow, Glasgow G31 2ER, UK; 19 Department of Obstetrics, Gynecology & Reproductive Sciences, University of Pittsburgh, Pittsburgh, PA 15261, USA; 20 Department of Oncology, University Hospital, Azienda Sanitaria Universitaria Friuli Centrale Udine, 33100 Udine, Italy; 21 Mayo Clinic, Phoenix, AZ 85054, USA; 22 Melbourne IVF, East Melbourne, VIC 3002, Australia; 23 Department of Surgery, Faculty of Medicine, Dentistry & Health Sciences, The University of Melbourne, Parkville, VIC 3052, Australia; 24 Department of Surgery, The Royal Melbourne Hospital, Parkville, VIC 3050, Australia; 25 Department of Medical Oncology, Peter MacCallum Cancer Centre, Melbourne, VIC 3000, Australia; 26 Sir Peter MacCallum Department of Oncology, Faculty of Medicine, Dentistry & Health Sciences, The University of Melbourne, Parkville, VIC 3052, Australia; 27 Sydney Children’s Hospital, Randwick, NSW 2031, Australia; 28 Academic Group and Children’s Cancer Institute, The University of New South Wales, Kensington, NSW 2052, Australia; 29 Musculoskeletal Health and Wiser Health Care, School of Public Health and Preventive Medicine, Monash University, Clayton, VIC 3168, Australia; 30 Dana-Farber Cancer Institute, Boston, MA 02215, USA; 31 Melbourne Medical School, The University of Melbourne, Parkville, VIC 3052, Australia; 32 College of Health, Medicine and Wellbeing, The University of Newcastle, Callaghan, NSW 2308, Australia; 33 Breast Cancer Network Australia, Camberwell, VIC 3124, Australia

**Keywords:** breast cancer, treatment-related amenorrhea, machine learning, cross imputation, risk prediction model

## Abstract

Treatment-induced ovarian function loss is a significant concern for many young patients with breast cancer. Accurately predicting this risk is crucial for counselling young patients and informing their fertility-related decision-making. However, current risk prediction models for treatment-related ovarian function loss have limitations. To provide a broader representation of patient cohorts and improve feature selection, we combined retrospective data from six datasets within the FoRECAsT (In**f**e**r**tility aft**e**r **Ca**ncer Predic**t**or) databank, including 2679 pre-menopausal women diagnosed with breast cancer. This combined dataset presented notable missingness, prompting us to employ cross imputation using the k-nearest neighbours (KNN) machine learning (ML) algorithm. Employing Lasso regression, we developed an ML model to forecast the risk of treatment-related amenorrhea as a surrogate marker of ovarian function loss at 12 months after starting chemotherapy. Our model identified 20 variables significantly associated with risk of developing amenorrhea. Internal validation resulted in an area under the receiver operating characteristic curve (AUC) of 0.820 (95% CI: 0.817–0.823), while external validation with another dataset demonstrated an AUC of 0.743 (95% CI: 0.666–0.818). A cutoff of 0.20 was chosen to achieve higher sensitivity in validation, as false negatives—patients incorrectly classified as likely to regain menses—could miss timely opportunities for fertility preservation if desired. At this threshold, internal validation yielded sensitivity and precision rates of 91.3% and 61.7%, respectively, while external validation showed 92.9% and 60.0%. Leveraging ML methodologies, we not only devised a model for personalised risk prediction of amenorrhea, demonstrating substantial enhancements over existing models but also showcased a robust framework for maximally harnessing available data sources.

## 1. Introduction

Breast cancer is one of the most frequently diagnosed cancers among women worldwide, accounting for 1 in 4 cancer cases and totalling 2.3 million instances in 2022 [1]. Globally, more than 100,000 women under the age of 40 are diagnosed with breast cancer each year [2]. Premenopausal patients with breast cancer receiving modern curative-intent treatment regimens, often including chemotherapy, have excellent long-term outcomes [3,4]. However, chemotherapy poses a risk of a loss of ovarian function and may result in early menopause or premature ovarian insufficiency (POI) [5,6,7,8]. This commonly presents as hypergonadotropic hypogonadism, resulting in amenorrhea (the cessation of menstrual cycles) [9]. The risk of treatment-related amenorrhea (termed “amenorrhea” hereafter) varies widely depending on factors such as age, chemotherapy regimen, and pretreatment ovarian reserve [10]. For example, most women aged 35–39 are at intermediate risk (31–58%) of treatment-related amenorrhea, with the risk increasing sharply with age—rising to approximately 77% in women over 40—depending on the chemotherapy regimen [11]. Many modern chemotherapy regimens are less damaging to ovarian function than older treatments, but they can still result in short- and long-term ovarian dysfunction [12,13]. Women who remain amenorrhoeic for 12 months following cytotoxic treatment are likely to experience permanent cessation of menstruation and a loss of reproductive capacity (i.e., infertility) [14,15]. The use of gonadotropin-releasing hormone agonists (GnRHa) during chemotherapy has been shown to reduce the risk of POI by temporarily suppressing ovarian function and thereby protecting ovarian reserve [16,17]. However, the evidence is not consistent [18,19].

The impact of ovarian function loss and infertility can be profound. Nearly 70% of young women diagnosed with early breast cancer express a desire to have children in the future [20,21]. Infertility often leads to substantial psychological distress, with levels of depression double that of the general population and diminished quality of life observed in areas such as emotional well-being, sexuality, and relationships [22]. The prospect of infertility can be a distressing outcome of such treatments [23]. Even for individuals without immediate plans for children, the threat of infertility can evoke feelings of anger and a sense of loss [22,24]. Concerns about infertility and the inability to conceive in the future may influence treatment decisions, leading some patients to opt for less effective cancer treatments or to not adhere to recommended endocrine therapy to preserve their fertility [25,26,27]. Providing accurate, personalised risk predictions can help patients better manage expectations and lead to greater satisfaction with their treatment-related decisions and reduced regret [28]. This may contribute to improved long-term psychosocial outcomes and an enhanced quality of life, even for women who remain childless [29,30,31,32]. Consequently, predicting the risk of developing amenorrhea after breast cancer treatment, prior to initiating gonadotoxic treatment, has emerged as an important area in current breast cancer research.

With the rapid proliferation of medical data and the advancement of modern statistical techniques and information technology, the utilisation of data science in constructing risk prediction models is a prominent area of research. Traditional risk prediction models, typically based on prior hypothesised knowledge, often consider the relationships between dependent variables. In contrast, machine learning (ML) methods have the potential to learn data models spontaneously, without requiring any implicit assumptions, and are capable of handling interdependence and complicated relationships between variables [33]. ML techniques excel in addressing the vast number of complex higher-order interactions present in medical data. Therefore, risk prediction models developed using ML methods have a high potential for application in clinical practice. An increasing number of clinical studies have leveraged ML methods to develop prediction models, which have found applications in diagnosis [34,35,36], disease risk prediction [37], and disease recurrence forecasting [38]. Despite increasing interest in predicting the risk of amenorrhea in young women diagnosed with breast cancer [39], existing studies often face significant limitations. This includes small datasets [40,41,42,43,44,45,46,47,48], exclusion of cases with missing data [40,41,42,43,44,45,46,47,48,49,50,51], reliance on a limited number of selected features [40,41,42,43,44,45,46,47,48,49,50,51], a focus on associations rather than predictions [52,53,54,55], incomplete reporting [40,41,42,43,44,45,46,47,48,49,50,51], and the absence of external validation [40,41,42,43,44,45,46,47,48,50]. To address these gaps, this study establishes a framework for applying machine learning methods in oncofertility research for amenorrhoea risk prediction. Specifically, we developed a predictive model combining multiple datasets to estimate the risk of amenorrhea 12 months after starting the gonadotoxic treatment in women with breast cancer, achieving improved discrimination and calibration.

The remainder of this manuscript is organised as follows. Section 2 describes the methodology, including data sources, cohort design, outcome definitions, and analytical procedures such as data cleaning, cross imputation, model development, internal and external validation, and model comparison. Section 3 presents the results of model building, associated variables, internal and external validation performance, and comparative analyses with existing prediction models. Section 4 discusses the findings in the context of previous research, highlights the strengths and limitations of our study, and outlines potential future work. Section 5 provides the main conclusions and implications for clinical practice and future research.

## 2. Materials & Methods

### 2.1. Cohort Design, Outcome Definition, Features, and Variables

The FoRECAsT (In**f**e**r**tility aft**e**r **Ca**ncer Predic**t**or) databank [56] comprises multiple datasets sourced from various national and international studies, including those from Australia, the UK, USA, Hong Kong, France, Denmark, Italy, Belgium, and International Trial Groups. In this research, menstrual status was collected at the beginning of chemotherapy. The outcome of amenorrhea was defined as the absence of menses for at least three consecutive months immediately preceding the 12-month time point after treatment initiation, irrespective of any subsequent resumption of menses. Intermittent bleeding or spotting within this 3-month window was treated as menses, and patients receiving hormonal suppression therapy were classified according to their menstrual status under treatment. Patients were considered premenopausal if they had their last menstrual period within the previous 12 months of treatment initiation/diagnosis.

From the FoRECAsT databank, seven datasets were selected and combined for analysis because they included the outcome variable amenorrhoea at 12 months from the initiation of treatment. Amenorrhoea at 12 months was a binary indicator, where 1 denoted the presence of amenorrhea and 0 indicated its absence. Among the seven selected datasets, six (‘A’, ‘D’, ‘E’, ‘F’, ‘G’, and ‘N’) were combined for model building and internal validation. The remaining dataset (‘M’), which was collected separately in a different research setting, was designated for external validation, as this configuration—using ‘M’ as the external validation set—resulted in the best model performance compared to other possible dataset divisions.

In this study, a feature referred to a non-outcome column in a dataset. Notably, a categorical feature could generate multiple variables, whereas a numerical or binary feature yielded only one variable. For numerical or binary features, the two terms ‘feature’ and ‘variable’ could be used interchangeably. In the selected datasets, not all features were utilised since some of them were not relevant. Only relevant features, selected based on domain knowledge, were incorporated in the data analysis.

### 2.2. Data Cleaning, Missing Values, and Cross Imputation

In the combined dataset, some features contained invalid values, which were identified using an internal data dictionary specifying valid ranges for each feature. Invalid values were corrected when obvious errors were present (e.g., typographical mistakes) or converted to missing values if no reliable correction was possible. For example, the feature “Smoking status” is coded as 0 (non-smoker) or 1 (smoker), whereas an invalid value of 13 was set as missing. Although our procedure is straightforward, proper handling of invalid values is crucial, as it can introduce bias into downstream analyses [57].

Additionally, missing values in the combined dataset arose from missing data at the time of collection, and variations in research settings across different datasets, leading to misalignment of certain features when combined and significant missingness. This misalignment meant that certain features existing in one dataset may be entirely missing in another.

To address these missing values, we employed cross imputation [58], a technique whereby missing values in one dataset are imputed using values from other datasets. This was accomplished using the k-nearest neighbours (KNN) algorithm and set the value of k to 10, meaning missing values were estimated based on the 10 closest neighbours in a multi-dimensional space. This approach is found to be effective in handling large missingness [59]. Remarkably, even in cases of very high missingness (>80%), it could still yield predictive models comparable to those trained with complete datasets [58,60]. The imputation process was facilitated using the R package recipes v1.1.1 [61], ensuring that as many records as possible were utilised to develop the predictive model.

### 2.3. Model Building, Internal Validation, and Calibration

Lasso regression, utilising a binomial family, was pivotal in constructing the model. This method was chosen for its interpretability and its ability to perform both variable selection and regularization, thereby maximising the log-likelihood while minimising the sum of the absolute values of regression coefficients and automatically removing unnecessary variables [62]. The R package glmnet v4.1-10 [63] was utilised for model construction.

**Figure 1:**
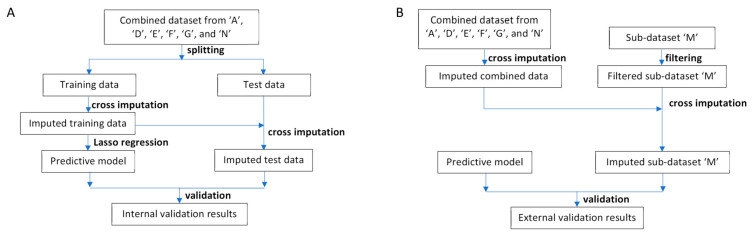
Data analysis workflow. (**A**) Workflow for model building and internal validation. First, the combined dataset was randomly split into training and test datasets. Cross-imputation was then conducted on the training data. Next, a predictive model was built using Lasso regression. Subsequently, cross-imputation was applied to the test data utilising the imputed training data. Finally, the model’s performance was evaluated. (**B**) Workflow for external validation. Initially, cross-imputation was performed on the combined dataset. Features exclusive to sub-dataset ‘M’ were omitted. Subsequently, cross-imputation was applied to the filtered sub-dataset ‘M’ using the imputed combined dataset. Finally, the predictive model’s performance was evaluated.

Model building and internal validation involved two phases: (i) feature selection and (ii) final model building. In the feature selection phase, the combined dataset was randomly split into training data and test data in 1:1 ratio. A predictive model was built from the imputed training data and was applied to the imputed test data to get the internal validation results (Figure 1A). To mitigate randomness, this process was repeated 100 times, with unnecessary variables automatically pruned in each iteration. Features were selected if at least one of their corresponding variables demonstrated significant relevance (*p*-value < 0.05) and appeared at least 50 times across the 100-round repetition.

In the final model building step, following feature selection, the process was further repeated 100 times. The final model’s coefficients were calculated from the mean values obtained across the 100 repetitions in this step. All internal validation results from the 100 repetitions were combined to yield the final internal validation results. Model performance was evaluated using the area under the receiver operating characteristic curve (AUC). For calculating sensitivity and precision, the cutoff was derived by maximising the F score with β = 1.5.

Calibration of predicted risks was performed as follows. The predicted risks of internal validation were divided into 20 percentage ranges (5% each) based on their values from smallest to largest. The calibrated predicted risk for each range was calculated as the percentage of cases falling within that range.

### 2.4. External Validation and Model Comparison

The external validation framework is illustrated in Figure 1B. Initially, cross imputation was carried out on the combined dataset. Subsequently, features unique to dataset ‘M’ that were absent from the combined dataset were excluded, as these features were not utilised during cross imputation or validation. Following this, cross imputation was applied to the dataset ‘M’ using the imputed combined dataset. Finally, our model was validated using the imputed dataset ‘M’.

To provide contextual reference for our model’s performance, we compared it with five previously published models [40,46,49,52,55] that reported sufficient details—specifically, the coefficients of associated variables—allowing application to our dataset. For each study, the published coefficients were applied to the imputed dataset ‘M’ and evaluated against our model’s external validation. While our model used a larger set of variables than these published models, the imputed dataset ‘M’ was generated using patterns from the combined training data. Therefore, applying published model coefficients to ‘M’ may artificially reduce their apparent performance, and these comparisons are intended to be exploratory rather than definitive head-to-head evaluations. Additionally, we compared our model with models reconstructed using the imputed combined dataset and the same associated variables and methods reported in each study. Some other studies [53,54] were excluded from such comparisons because their variables were not all available in the imputed dataset ‘M’, making direct evaluation infeasible.

## 3. Results

### 3.1. Study Participants and Missing Values

The seven selected datasets (‘A’, ‘D’, ‘E’, ‘F’, ‘G’, ‘M’, and ‘N’) from the FoRECAsT databank comprised 3795 individual patient records. Of these, 962 records (25.3%) were excluded due to missing outcome data. The remaining 2833 individual patient records were used for model development and validation. Following data cleaning and preparation, these records were subjected to feature selection and modelling to construct the amenorrhoea risk prediction model. Detailed summary statistics for the selected datasets can be found in Table 1.

The model construction and validation dataset comprised 6 datasets (‘A’, ‘D’, ‘E’, ‘F’, ‘G’, and ‘N’) containing 2679 individual patient records with 53 features, reflecting an amenorrhea prevalence rate of 48.3%. The combined dataset exhibited 61.4% missingness (Table 1). The missing rate for each feature is detailed in Supplementary File S1, while the explanations of these features can be found in Supplementary File S2.

### 3.2. Model Development and Associated Variables

Sixty-two variables from 53 features are summarised in Supplementary File S3. During feature selection, twenty-two variables from 20 features had significant relevance (*p*-value < 0.05) and appeared at least 50 times in the 100 rounds of repetition. Consequently, these 20 features were selected for the final model building phase, which underwent another 100 rounds of repetition. In this phase, two features, namely “Contraception” and “Radiotherapy treatment”, were further excluded due to their corresponding variables appearing less than 50 times. In the final model, 20 variables from 18 features, along with the intercept value, were utilised to predict amenorrhea at 12 months after chemotherapy initiation (Table 2). The average coefficients from the 100-round repeats were used for the final model. The 95% confidence intervals were calculated from the empirical distribution of the coefficients across 100 repeats (using the 2.5th and 97.5th percentiles) to approximate the uncertainty. For numeric variables, the adjusted coefficient was derived by multiplying the coefficient by one standard deviation (SD) of that variable (calculated after imputation). The adjusted odds ratio (OR) represents the change in odds associated with a one-SD increase in the variable, while the unadjusted OR represents the effect per unit increase. The coefficients in their original scale are retained for clinical interpretability, and the adjusted coefficients provide a standardised comparison across variables. Utilising the coefficient values of the final model, we can predict a patient’s risk of amenorrhea and calculate the odds using the following formulas:

$$probability=\;\frac{1}{1+e^{-\left(\beta_{0}+\beta_{1}x_{1}+\beta_{2}x_{2}+\ldots +\beta_{20}x_{20}\right)}}\;odds=\frac{probability\;of\;event}{1-probability\;of\;event}$$

Our model identified 20 variables associated with amenorrhea (Table 2). According to our findings, the *BRCA* pathogenic variants emerged as the leading predictor of amenorrhea. *BRCA2* carriers exhibited 356% higher odds compared to *BRCA* non-carriers, while *BRCA1* carriers showed 74% higher odds compared to *BRCA* non-carriers. Another significant variable was the number of treatment cycles involving a combination of adriamycin, cyclophosphamide, and methotrexate, fluorouracil (AC+CMF cycles), with each additional cycle associated with 68% higher odds. Furthermore, each additional year of age was associated with a 6% increase in odds. For a comprehensive overview of our findings, please refer to Table 2.

### 3.3. Model Evaluation

**Figure 2:**
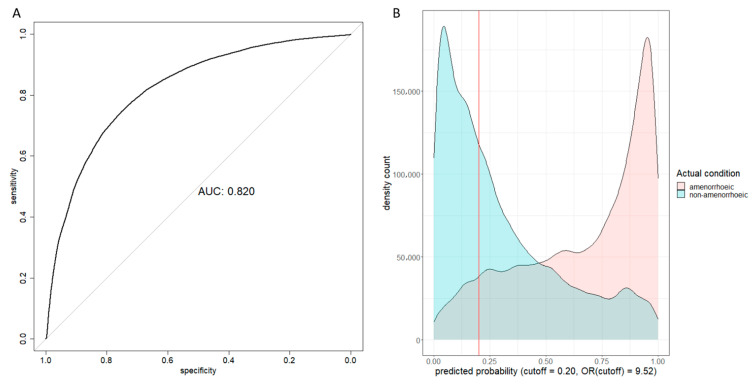
Internal validation results. (**A**) Receiver operating characteristic curve and area under curve (AUC) plot; (**B**) Predicted probability density count plot. The OR (cutoff) was the odds ratio between the two patient groups on two sides of the cutoff line.

During internal validation, the AUC was 0.820 (95% CI: 0.817–0.823) (Figure 2A). Given the objective of prioritizing sensitivity over precision in predicting amenorrhea, the F score with β = 1.5 was maximised to derive a cutoff value of 0.20. As a result, the sensitivity reached 91.3% while precision stood at 61.7%. Figure 2B provides a clear visualisation, indicating that most patients experiencing amenorrhea at 12 months after treatment initiation fell to the right of the cutoff threshold. Details of the calibrated predicted risks can be found in Table 3.

For external validation, the independent dataset ‘M’ was employed, comprising 154 patients and 13 features, with the prevalence of amenorrhea being 54.5% and 27.1% missingness (Table 1). All features present in the dataset ‘M’ were also included in the combined dataset (Supplementary File S4). Notably, age was the only feature available in dataset ‘M’ directly overlapped with the 18 modelling features from the final model (Table 2 and Supplementary File S4). The remaining required features were cross-imputed from the 13 available features in ‘M’, using patterns learned from the combined training datasets. This approach allowed us to evaluate the feasibility of applying the model to an external dataset with partially missing or misaligned predictors—a common challenge in multi-cohort oncofertility research. Although this form of validation does not constitute a fully independent assessment, it demonstrates how the model and imputation framework can be applied jointly to harmonise heterogeneous datasets. Following this procedure (Figure 1B), we obtained an AUC of 0.743 and calculated a 95% CI of 0.666–0.818 using bootstrapping. Employing a cutoff of 0.20, the sensitivity and precision were determined as 92.9% and 60.0%, respectively. A lower cutoff of 0.20 was selected to achieve higher sensitivity, as false negatives—patients incorrectly classified as likely to regain menses—potentially have more profound impact than false positives due to missed opportunity for timely fertility preservation for those who wish to pursue it.

**Figure 3:**
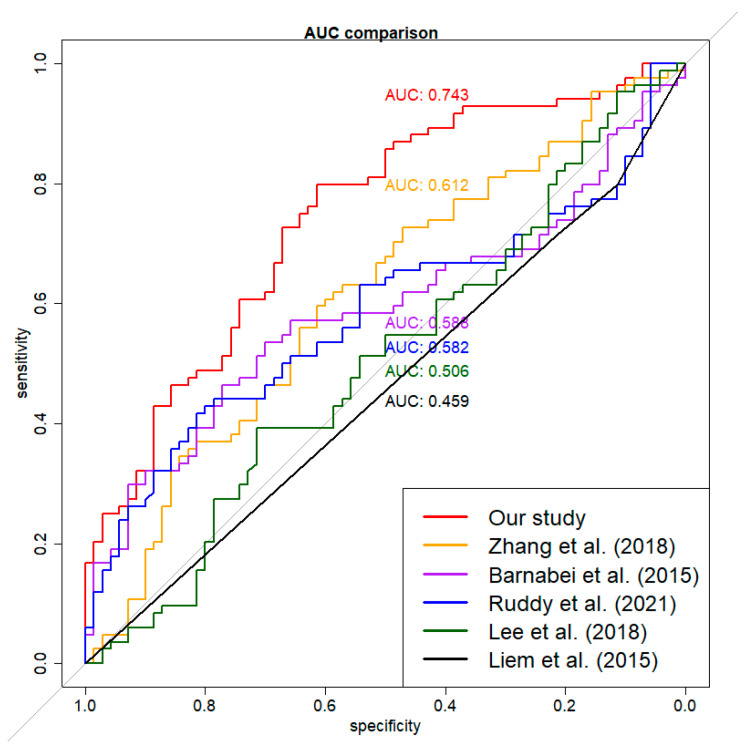
Exploratory AUC performance comparison for six models. The imputed external dataset was used for validation. The red curve was for our model. The other curves were for models built using the imputed combined dataset on the same associated variables and methods of previously published studies [40,46,49,52,55]. Differences in variable definitions and imputation mean these results are for contextual reference only.

For contextual reference, our model’s performance was assessed alongside five previously published models [40,46,49,52,55]. When the published coefficients were applied to the imputed dataset ‘M’, the corresponding AUCs were 0.459, 0.506, 0.540, 0.600, and 0.610, respectively. Models reconstructed using the same variables and methods on the imputed combined dataset yielded AUCs of 0.459, 0.506, 0.582, 0.588, and 0.612 (Figure 3). Because the imputed dataset ‘M’ was generated using patterns from our training data, these AUCs may underestimate the true performance of the published models. Therefore, these comparisons are exploratory and should not be interpreted as evidence of superiority. The primary focus remains on the performance of our model and the relevance of its selected features.

## 4. Discussion

We developed and externally validated a machine learning model to accurately predict treatment-related amenorrhea in young women with breast cancer 12 months after chemotherapy initiation. The model achieved strong discrimination in both internal and external validation and was optimised for high sensitivity to minimise missed opportunities for fertility preservation, a priority in clinical decision-making for this population. This is the largest and most diverse dataset used for this outcome to date, with the integration of six international datasets from the FoRECAsT databank combined with robust methods to handle substantial missingness and select relevant features, produced a model that demonstrated consistent performance when applied to an independent dataset.

A key strength of this work is the integration of multiple datasets from diverse clinical settings, encompassing wide variation in patient characteristics, treatment regimens, and follow-up patterns. This diversity created a more representative and comprehensive feature space than in previous studies. Previous studies focused on identifying a limited number of variables associated with amenorrhea or building predictive models with a handful of features. However, constructing a precise amenorrhea risk prediction model demands careful consideration of all potentially relevant features. Our study was the first to screen many potentially relevant features to build a predictive model. In contrast to conventional logistic regression approaches, our study leveraged Lasso regression to construct the model. This choice allowed for the automatic selection of pertinent predictors, and effective mitigation of overfitting while preserving the model’s interpretability. Importantly, we deliberately avoided black-box approaches, as model interpretability is essential in healthcare settings to ensure transparency, trust, and reduced bias [64]. The robustness of our approach is evident from its consistent external validation performance, with exploratory comparisons to five previously published studies [40,46,49,52,55]. This study is a part of the broader FoRECAsT research project, with the goal of developing a web-based clinical predictive tool for amenorrhea risk, similar to predicting the survival rates after surgery with different treatment combinations for young women with breast cancer [65].

The findings from this study should be interpreted with caution, as the 12-month amenorrhoea outcome from the chemotherapy initiation does not necessarily reflect long-term implications. Some women may regain menses later if sufficient primordial follicles remain in the resting pool. In contrast, others may experience delayed amenorrhoea beyond this point as ovarian reserve continues to decline over time. Our aim in this study was to demonstrate how different clinical datasets can be compiled and utilised—with imputation—within this modelling framework for future risk prediction. Our subsequent publications will provide further insight by reporting long-term amenorrhoea outcomes over a 12–60-month period.

In modelling, different studies may employ various ML algorithms and feature selections, making direct comparisons of coefficient values for shared variables challenging. Instead, the focus should be on evaluating the relevance of features and their impact on risk. Liem et al. (2015) [55] identified only older age as a risk factor for amenorrhea, with BMI, smoking, chemotherapy, and trastuzumab showing no significant relevance. Zhang et al. (2018) [49] found that older age, lower E2, and higher FSH were associated with increased risk, while BMI was not relevant. Ruddy et al. (2021) [52] suggested that older age, lower BMI, and lower AMH increased risk, with chemotherapy and tamoxifen showing no relevance. Poorvu et al. (2021) [54] indicated that older age, lower BMI, tamoxifen, and chemotherapy were linked to increased risk, with smoking showing no significant relevance. Kabirian et al. (2023) [53] identified older age, hot flashes at diagnosis, endocrine therapy, and trastuzumab as factors increasing risk, with BMI and smoking showing no significant relevance. These findings indicate that the associated variable sets found in previous studies do not always match. In our study, we examined 53 potential features and identified factors such as *BRCA* mutations, a higher number of AC+CMF cycles, older age, lower E2, lower AMH, and higher FSH as contributors to increased amenorrhea risk (Table 2). In contrast, factors such as lower BMI, smoking, trastuzumab, and tamoxifen were considered during feature selection but were excluded from the final model due to their limited importance (Supplementary File S3). Overall, our study’s findings regarding features associated with amenorrhea align with most of those reported in previous studies, providing consistency and further insight into the predictors of amenorrhea.

Direct performance comparison with previously published models is limited due to differences in variable definitions, population characteristics, and data contexts. In particular, because the imputed dataset ‘M’ was generated using patterns from our combined training data, applying coefficients from the literature models may artificially reduce their apparent AUCs. Accordingly, these comparisons are presented as exploratory references rather than definitive evaluations. Nonetheless, feature-level comparisons remain meaningful: across studies, older age, lower AMH, and higher FSH consistently predicted increased risk of amenorrhea, supporting the biological plausibility of our findings. Our study further identified *BRCA* mutations and the number of AC+CMF cycles as strong predictors, reflecting the broader variable set examined and highlighting the additional insight gained by incorporating more variables in our model.

Cross imputation played a pivotal role in the development and application of our model. Previous studies have taken varied approaches to handling missing values. Some studies did not explicitly address missing values [55], while others treated them as a separate category [49] or removed records with missing values entirely [52]. Another study predicting primary ovarian insufficiency after chemotherapy used multiple imputations by chained equations (MICE) to handle missing values, assuming data were missing at random [66]. However, given the nature of our combined dataset, treating missing values in the same manner as these studies was not feasible. Instead, we employed cross imputation, which allowed us to generate a fully imputed dataset comprising 2679 samples with 53 features for constructing our model. These 53 features include 18 modelling features utilised by our model and 35 non-modelling features. When applying our model to an external dataset, it is plausible that the external dataset may lack some modelling features while containing some non-modelling features. Although the non-modelling features do not contribute to prediction directly, they are utilised for imputing modelling features in the external dataset during cross imputation (Figure 1B). This approach enables our model to be applied to external datasets collected in diverse research settings. The external validation conducted with the dataset ‘M’ underscored the robustness of our approach and demonstrated the strength of our study. Furthermore, this cross-imputation approach holds promise for adaptation by other medical researchers seeking to build predictive models in similar contexts.

### Limitations

This study has several limitations. First, despite being the largest dataset assembled to date for this outcome, some contributing datasets were small (e.g., 96 to 154 records) and showed marked variation in amenorrhea prevalence (10.8 to 78.1%), which may introduce bias. Secondly, the model was externally validated on a single independent dataset; broader validation across multiple independent and prospective cohorts where there are primary data on the selected features is needed to confirm generalisability. Thirdly, two chemotherapy-related variables (*CMF dose* and *CMF treatment*) showed an inverse association with amenorrhea, contrary to clinical expectations, likely reflecting multicollinearity with other chemotherapy measures or dataset heterogeneity; these should be interpreted within the context of the full model. Fourthly, while the model was reduced to 18 features to balance accuracy and feasibility, some settings may still require fewer inputs. Additionally, although our model underwent external validation using dataset ‘M’, most modelling features were cross-imputed rather than directly observed. Consequently, this procedure reflects the performance of the combined imputation–prediction framework rather than purely independent validation. Finally, our outcome—amenorrhea at 12 months after chemotherapy initiation, does not fully capture long-term ovarian function; some women may recover menses later, while others may develop delayed amenorrhea.

## 5. Conclusions

We presented a robust, interpretable, and externally validated machine learning model for predicting treatment-related amenorrhea in young women with breast cancer. By integrating diverse datasets and applying rigorous imputation and feature selection methods, our model incorporating 20 established and novel variables achieved strong predictive performance in both internal and external validation.

Future work will focus on several directions. First, extending the prediction horizon to 12–60 months will allow assessment of longer-term ovarian function outcomes for young breast cancer patients. Secondly, we will pursue further validation across larger, prospective, and international cohorts, including testing the model on external datasets with directly measured predictors or simplified versions of the model using only universally available features to evaluate true transportability and generalisability. Thirdly, alternative interpretable and non-interpretable machine learning algorithms (e.g., elastic net, random forest, or gradient boosting) will be explored to assess potential performance improvements. Finally, integrating this model into a freely available web-based clinical tool will facilitate personalised fertility risk prediction at different timepoints across survivorship, and support shared decision-making for young women receiving gonadotoxic treatment.

Together, this framework provides a scalable foundation for precision oncofertility and can be adapted for other clinical prediction applications where data heterogeneity and missingness present challenges.

## Figures and Tables

**Table 1:** Summary of the selected sub-datasets, after excluding records with missing outcome values.

	Observations n	Total Feature n	Numerical Features n	Binary Features n	Categorical Feature n	Prevalence of Amenorrhea at 12 Months %	Data Missingness %
A	280	26	7	16	3	48.9	6.6
D	725	11	3	8	0	10.8	10.2
E	209	22	10	9	3	21.1	11.8
F	96	28	10	16	2	78.1	15.7
G	101	19	6	9	4	40.6	10.9
M	154	13	4	7	2	54.5	27.1
N	1268	27	10	12	5	72.5	13.9
Total	2833	53	23	22	8	48.6	62.0
ADEFGN combined	2679	53	23	22	8	48.3	61.4

**Table 2:** Summary of the model’s coefficients. The adjusted coefficient for numeric variables was calculated by multiplying the coefficient by one standard deviation (SD) of the variable; for categorical variables, it remains the same as the coefficient. The odds ratio (OR) and adjusted OR represent the change in the predicted probability of Amen_ST12 for a one-unit and one-SD change in the variable, respectively. The 95% confidence intervals were estimated from the 2.5th and 97.5th percentiles of the coefficients across 100 repeats. The table is sorted by the absolute value of the adjusted coefficients, from largest to smallest.

Order	Variable	Coefficient	OR	Adjusted Coefficient	Adjusted OR [95% CI]
	Intercept	5.193			
1	*BRCA2*	1.516	4.56	1.516	4.56 [4.024, 5.158]
2	*BRCA1*	0.554	1.74	0.554	1.74 [1.657, 1.829]
3	AC+CMF cycles	0.519	1.68	0.553	1.74 [1.589, 1.903]
4	CMF dose	−7.329 × 10^−4^	1.00	−0.483	0.62 [0.586, 0.650]
5	No Chemotherapy doses	−0.443	0.64	−0.443	0.64 [0.615, 0.670]
6	Taxanes dose	0.004	1.00	0.403	1.50 [1.406, 1.592]
7	CMF treatment	−0.385	0.68	−0.385	0.68 [0.649, 0.714]
8	Age	0.060	1.06	0.384	1.47 [1.432, 1.506]
9	CMF cycles	0.204	1.23	0.335	1.40 [1.342, 1.456]
10	Inhibin B	−0.017	0.98	−0.321	0.73 [0.685, 0.769]
11	Cycles of other chemotherapy	−0.676	0.51	−0.301	0.74 [0.721, 0.759]
12	AFC	−0.052	0.95	−0.276	0.76 [0.688, 0.837]
13	Chemotherapy dose per 3 weeks	0.228	1.26	0.228	1.26 [1.192, 1.325]
14	AMH	−0.036	0.96	−0.204	0.82 [0.783, 0.850]
15	Estradiol	−7.582 × 10^−5^	1.00	−0.195	0.82 [0.804, 0.841]
16	Neoadjuvant Chemotherapy	0.173	1.19	0.173	1.19 [1.159, 1.219]
17	Total doses per mg	6.522 × 10^−5^	1.00	0.116	1.12 [1.107, 1.138]
18	FSH	0.007	1.01	0.115	1.12 [1.097, 1.148]
19	LH	0.010	1.01	0.102	1.11 [1.094, 1.121]
20	Locoregional radiotherapy	0.100	1.11	0.100	1.11 [1.058, 1.155]

*BRCA2*: BReast CAncer gene 2 mutation, *BRCA1*: BReast CAncer gene 1 mutation, AC: Adrimyacin and Cyclophosphamide, CMF: Cyclophosphamide, Methotrexate, and Fluorouracil, AMH: Anti-Müllerian Hormone, AFC: Antral Follicle Count, FSH: Follicle-Stimulating Hormone, LH: Luteinising Hormone. Note: The effect for *BRCA2* and *BRCA1* were compared to *BRCA* non-carriers; the effect for “No Chemotherapy doses” and “Chemotherapy dose per 3 weeks” were compared to “Chemotherapy dose per 2 weeks”.

**Table 3:** The list of predicted probability, percentage range, and calibrated predicted probability.

Predicted Probability %	Percentage Range %	Calibrated Predicted Probability %
[0.00, 2.75)	[0.0, 5.0)	8.9
[2.75, 5.73)	[5.0, 10.0)	8.6
[5.73, 9.18)	[10.0, 15.0)	12.7
[9.18, 13.07)	[15.0, 20.0)	16.6
[13.07, 16.73)	[20.0, 25.0)	20.4
[16.73, 21.09)	[25.0, 30.0)	22.5
[21.09, 25.42)	[30.0, 35.0)	28.8
[25.42, 30.71)	[35.0, 40.0)	32.6
[30.71, 36.67)	[40.0, 45.0)	38.0
[36.67, 43.31)	[45.0, 50.0)	44.5
[43.31, 50.54)	[50.0, 55.0)	49.7
[50.54, 57.79)	[55.0, 60.0)	55.9
[57.79, 65.60)	[60.0, 65.0)	61.8
[65.60, 73.57)	[65.0, 70.0)	66.7
[73.57, 80.54)	[70.0, 75.0)	74.3
[80.54, 86.06)	[75.0, 80.0)	75.6
[86.06, 90.44)	[80.0, 85.0)	80.2
[90.44, 94.03)	[85.0, 90.0)	86.6
[94.03, 96.99)	[90.0, 95.0)	89.1
[96.99, 100.00]	[95.0, 100.0]	89.1

## Data Availability

The data presented in this study are available on request from the corresponding author due to ethical reasons.

## Supplementary Materials

The following supporting information can be downloaded at: https://www.mdpi.com/article/10.3390/bioengineering12111171/s1, Supplementary File S1: Summary of missingness in the combined dataset; Supplementary File S2: The explanation of all features used in this study; Supplementary File S3: All features derived in the first step of model development; Supplementary File S4: Summary of missingness in the dataset ‘M’.
